# Erratum: Attentional capture by fearful faces requires consciousness and is modulated by task-relevancy: a dot-probe EEG study

**DOI:** 10.3389/fnins.2023.1247546

**Published:** 2023-07-04

**Authors:** Frontiers Production Office

**Affiliations:** Frontiers Media SA, Lausanne, Switzerland

**Keywords:** spatial attention, awareness, fearful faces, EEG, mass univariate analysis, multivariate pattern analysis

**Text Correction**

Due to a production error, the word “behavioural” was replaced with “al” in the Abstract.

A correction has been made to the Abstract:

“In the current EEG study, we used a dot-probe task in conjunction with backward masking to examine the neural activity underlying awareness and spatial processing of fearful faces and the neural processes for subsequent cued spatial targets. We presented face images under different viewing conditions (subliminal and supraliminal) and manipulated the relation between a fearful face in the pair and a subsequent target. Our mass univariate analysis showed that fearful faces elicit the N2-posterior-contralateral, indexing spatial attention capture, only when they are presented supraliminally. Consistent with this, the multivariate pattern analysis revealed a successful decoding of the location of the fearful face only in the supraliminal viewing condition. Additionally, the spatial attention capture by fearful faces modulated the processing of subsequent lateralised targets that were spatially congruent with the fearful face, in both behavioural and electrophysiological data. There was no evidence for nonconscious processing of the fearful faces in the current paradigm. We conclude that spatial attentional capture by fearful faces requires visual awareness and it is modulated by top-down task demands.”

The publisher apologizes for this mistake. The original article has been updated.

